# Real-Time Regulation Model of Physical Fitness Training Intensity Based on Wavelet Recursive Fuzzy Neural Network

**DOI:** 10.1155/2022/2078642

**Published:** 2022-04-22

**Authors:** Zhaoxin Shen, Ying Yang

**Affiliations:** ^1^Beijing Sport University, Haidian, Beijing 100084, China; ^2^Beijing Foreign Studies University, Haidian, Beijing 100089, China

## Abstract

It has been widely used in signal processing, image processing, speech recognition and synthesis, pattern recognition, machine vision, machinery fault diagnosis and monitoring, and other scientific and technological fields and has achieved great results. The application potential in nonlinear system identification is increasing. According to the theory of “overload recovery” and “functional reserve”, the mathematical model of “load-fitness state” is established to understand the adaptation characteristics and individual characteristics of athletes to sports training. The model is used to simulate the values and time required to reach the maximum fitness state for four types of precompetition reduction plans and to provide a reference for the development of precompetition training plans. The data required for parameter estimation were the actual training data of six outstanding basketball athletes (mean age 18.2 ± 0.75, mean training years 4.6 ± 0.49). And the coaches' training plan was not intervened during the test. In order to further reduce the biaxial synchronization error of the sports platform and improve the stability of the system, the wavelet transformation capable of time-varying signal analysis and the recursive structure with dynamic capability were combined with the fuzzy neural network, and the learning ability of the neural network was used to learn and adjust the scaling and translation factors in the wavelet function, the mean and standard deviation in the fuzzy structure, and the connection weights between the layers, according to the biaxial synchronization. The simulation results show that the designed global sliding mode controller can improve the convergence speed of tracking error and ensure the single-axis tracking accuracy of the H-type motion platform compared with the traditional sliding mode controller, and the tracking accuracy and synchronization accuracy of the system can be further improved after adding the cross-coupled synchronization controller, but the improvement of synchronization control accuracy is not very satisfactory due to the fixed selection of the parameters of the cross-coupled controller. Further improvement is needed.

## 1. Introduction

The athlete must undergo a comprehensive and systematic physical fitness diagnostic assessment, including assessment of body composition, assessment of body part function, basic body function examination, and injury history examination [1]. It is also important to make the most effective assessment based on the individual athlete, which is fundamental to an effective training program [2]. It is also necessary to perform a systematic physical fitness assessment and diagnosis during the off-season and during the season so that we can always know the athletes' fitness status and provide basic reference information for future training [3]. As an important part of sports, the importance of professional and systematic sports training is obvious. Postinjury preventive fitness training is an integral part of overall fitness training and somehow determines the effect of basic and special fitness training [4]. In the framework of postinjury injury preventive fitness training system, contains its two main focus, one is the rehabilitation of physical training based on injury preventive fitness training; another is the active monitoring of its sports load. These two aspects are used to reduce the risk of injury and thus improve the athletes' performance [5]. In modern athletic training science, with the scientific training method, the ability of multiple sciences to intervene together is particularly important. For example, in the United States, as the first technical force and from the point of view of sports, sports have always focused on the physical practice training injury prevention physical training and scientific research combined to form a complete system and a system of theory and practice [6].

In USA Basketball, sports training does not exist singularly but as a whole in cooperation with multiple parties. The training program and the implementation of the training program are based on scientific research and clinical tests and are arranged according to the specific athletic conditions and physical characteristics of the athletes, including their athletic characteristics. Their physical training is divided into basic testing, basic assessment, setting training goals, setting training plans, and integrated implementation. Especially in the free exercises of auxiliary equipment, the coach pays great attention to the basis of these exercises. Even minor mistakes can be corrected in time [7]. They believe that only in this way can they gradually increase the training load, improve the effectiveness of training, and prevent injuries. Fuzzy neural networks, in addition to the computing and learning capabilities of general neural networks, can also combine the unique ability of fuzzy systems to approximate human knowledge expression and understanding, but when dealing with complex nonlinear system problems, processing by fuzzy neural networks alone is still insufficient [8]. The term wavelet was first introduced by Morlet and Grossman in the early 1980s. In terms of presentation, a wavelet is an oscillation whose amplitude increases from zero and ends at zero. In recent years, wavelet analysis has received a great deal of attention among engineers and mathematicians [9]. In contrast to Fourier analysis, wavelet analysis has two different mathematical theoretical bases, one is the wavelet integral transform, a convolution operation on some elementary wavelet function, and the other is the wavelet level, which uses a single wavelet function and represents it by adding a binary expansion and a translation of the integral [10].

Therefore, the recursive wavelet fuzzy neural network compensator designed by combining wavelet fuzzy neural network with recursive structure can have better dynamic characteristics and system response when unknown parameters, external disturbances, and parameters change [11]. In this paper, the model refers to the wavelet recurrent fuzzy neural network model, and the variation of the adaptation quantity in the model is improved nonlinearly by increasing the limit quantity parameter of adaptation. The data required for parameter estimation were the actual training data of six outstanding basketball athletes (average age 18.2 ± 0.75, average training years 4.6 ± 0.49), and the coaches' training program was not intervened during the test.

In order to further reduce the biaxial synchronization error of the sports platform and improve the stability of the system, the wavelet transformation capable of time-varying signal analysis and the recursive structure with dynamic capability were combined with the fuzzy neural network, and the learning ability of the neural network was used to learn and adjust the scaling and translation factors in the wavelet function, the mean and standard deviation in the fuzzy structure, and the connection weights between the layers, according to the biaxial synchronization. The recursive wavelet fuzzy neural network compensator is designed to replace the cross-coupled synchronization controller according to the two-axis synchronization error.

## 2. Physical Training Intensity Real-Time Regulation Model

### 2.1. Real-Time Monitoring of Physical Strength

**Figure 1:**
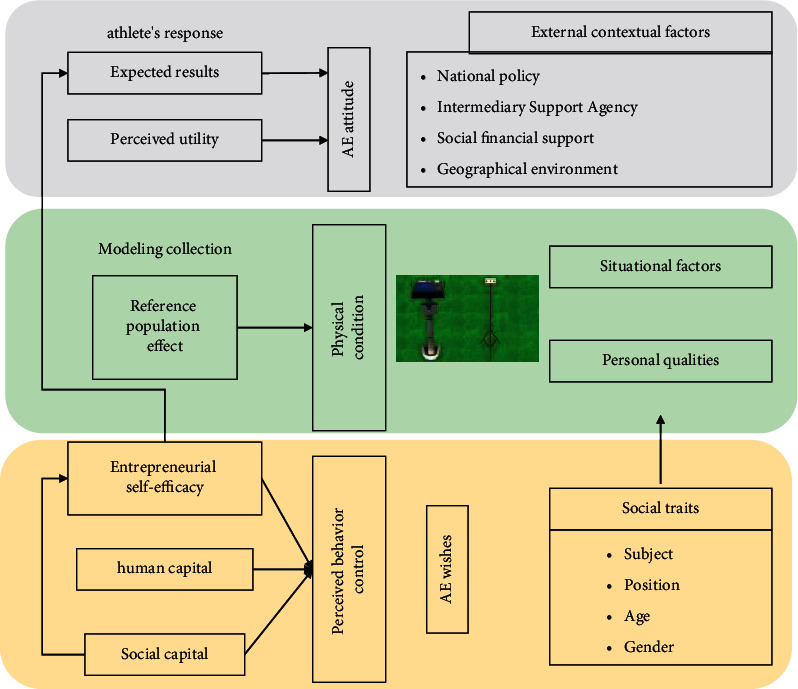
Real-time monitoring model of physical strength.

Physical fitness is one of the important factors in the composition of athletes' competitive ability, and the level of physical fitness can affect the play of techniques and tactics in the game and is an important guarantee of the athletes' technical and tactical execution ability [12]. How to improve the athletes' special physical ability is undoubtedly important to the improvement of athletes' competitive level. Among them, how to make the training plan for the adjustment period before the game so that the athletes can eliminate the fatigue and recover their physical ability to participate in the game is an important part of the physical training. The purpose of this paper is to investigate the process of using mathematical modeling to analyze the effects of training on athletic performance and to understand the individualized adaptive characteristics of athletes. In the process of modeling the system for predicting the athlete's response to training, it is necessary to simplify this training adaptation system. The simplification process includes the selection of the input and output variables of the system, the creation of the model structure, the collection of data for the identification of the model parameters, and the application of the identified model for prediction, as shown in Figure 1. In order to find the best model, mathematical and statistical methods are needed to compare the fitting ability of different models.

Banister et al.'s model assumes that exercise capacity is a balance between fatigue and adaptation, and their study shows that the model fits the exercise adaptation process well for subjects in laboratory conditions and for discus, weightlifting, distance running, and swimming athletes [13]. The ability to fit the model adequately demonstrates the validity of the theoretical assumptions supporting the model, which are important for enriching sports training theory, such as increasing the training load so that the increase in fatigue exceeds the increase in adaptation, resulting in a temporary decrease in athletic performance, and continuing to decrease the load so that the rate of decline in fatigue exceeds the rate of decline in adaptation, resulting in a temporary increase in athletic performance. Modeling of the training response of good swimmers has confirmed the above processes.

These findings provide a new explanation for the phenomenon of precompetition load reduction leading to an increase in athletic performance. In order to find the best model, mathematical and statistical methods are needed to compare the fitting ability of different models. For example, assuming that the amount of fatigue changes with the accumulation of training volume, a nonlinear model is built on this basis, and the newly established model statistically confirms a better fit to the data than the original model, which would explain well the phenomenon that athletes have difficulty adapting when training with loads that are beyond the norm. Conversely, when the load was reduced, it was easy to produce an improvement in athletic performance recovery. Modeling experiments conducted on nonathletes individually under laboratory conditions have the advantage of improving the confidence and discrimination of model parameter estimates, as shown in Table 1, but the method of estimating parameters under laboratory conditions is not applicable to modeling training adaptations of athletes under real field conditions. Based on the relevant model and parameters, it is possible to apply a computer to simulate the response of the physical state to a loading stimulus.

Although we already understand the relationship between the increase and decrease of sports training volume and athletic performance, the existing mathematical models do not have the ability to accurately predict the training process of a given athlete to monitor it precisely. The application of a systems theory approach to the study of responses to athletic training is becoming a hot topic of research. This research was initiated by Banister and colleagues, and their initial model has now taken several extended forms.

### 2.2. Online Target Tracking and Deep Learning Strategies

Methods of quantifying training load can be applied well under standard laboratory conditions, and these methods are simple quantification of a single training tool [14]. The situation becomes much more complex when quantifying load in field testing athletes because of the diversity of training means and content. The established quantification methods are performed by assigning different weights to different training means and intensities. In endurance sports, weights for different exercise intensities are determined using the percentage of heart rate reserve method, so that the quantification function to quantify the load is called training impulse, or TRIMP. The shortcomings of the training load quantification method must lead to limitations in the application of the model.

A multi-input modeling approach may be able to better simulate the process of exercise adaptation, and multiple inputs can summarize the content of various training forms in the training program, but this approach modeling is highly specific to the exercise program and requires more stringent accuracy in data collection because there are more variables and parameters in this model and more systematic errors. The experimental and observed data enable the structure of the model to be determined as well as the estimation of the model parameters. When the inputs and outputs are known, it is necessary to identify the model, that is, what kind of model can explain well the data obtained from observations.

Due to the complexity of the physiological process of training adaptation, the model can only be an abstraction of the main features of this complex systemic process, and it is impossible to consider all the influencing factors. Modeling the process of training adaptation can only consider the dynamic process of change of athletic ability during the change of training load because, for the athlete, sports training is the most important factor affecting athletic ability, as shown in Table 2. An important step in the analysis of mathematical models is to test how well the model fits the observed data, that is, how well the model describes the changes in the data.

**Figure 2:**
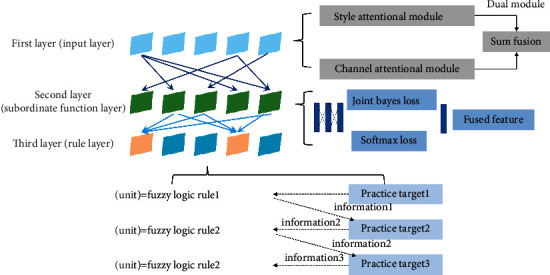
Recursive wavelet fuzzy neural network compensation controller structure.

As shown in Figure 2, the recursive wavelet fuzzy neural network is structurally divided into five layers, with three implicit layers, namely the affiliation function layer, the rule layer, and the recursive wavelet function layer [15]. The neural network controller combines fuzzy logic, wavelet processing, and recursive structure to improve its processing capability and accuracy and solve the shortcomings of static mapping.

#### 2.2.1. First Layer (Input Layer)

Both neuron nodes in this layer are input nodes, which are equivalent to the input variables. They are the two-axis position synchronization error *e* and the two-axis velocity synchronization error of the H-type motion platform, respectively. The linear transformation relationship between the input and output of neurons in this layer can be expressed as follows:

(1) $$\begin{array}{c} x_{i}=N_{i}^{l}\left(N\right),\\ y{\left(x\right)}_{i}=f_{i}\left(N_{i}^{l}\left(N\right)\right),\\ f_{i}\left(N_{i}^{l}\left(N\right)\right)={\text{net}}_{i}\left(N\right),\;i=1,2,\ldots , \end{array}$$

where *x*_*i*_ is the input signal of the input layer, and the input variables are the position synchronization errors, respectively.

(2) $$\begin{array}{c} x_{i}^{l}=\frac{e_{y1}-e_{y2}}{e\left(t\right)}. \end{array}$$

Speed synchronization error is as follows:

(3) $$\begin{array}{c} x_{1}^{l}=\frac{e_{y1}-e_{y2}}{e\left(1\right)},\\ x_{2}^{l}=\frac{e_{y1}-e_{y2}}{e\left(2\right)}, \end{array}$$

where *e*_1_ and *e*_2_ are the position tracking errors of the *Y*_1_-axis linear motor and *Y*_2_-axis linear motor, respectively; *y*_*i*_(*N*) is the output signal of the input layer; *N* is the number of sampling times.

#### 2.2.2. Second Layer (Subordinate Function Layer)

The output of each neuron in the input layer corresponds to 3 neurons in the affiliation function layer. The nonlinear transformation in the affiliation function layer uses a Gaussian function, and this transformation method incorporates a fuzzy logic inference approach to improve the inductive performance of the network. The linear transformation relationship between the input and output of neurons in this layer can be expressed as follows:

(4) $$\begin{array}{c} \text{net}\left(N\right)=\frac{{\left(x^{2}-m_{j}\right)}^{2}}{\sqrt{x^{2}-\tilde{x^{2}}}},\\ y^{2}\left(N\right)=f\left(\text{net}\left(N\right)\right)=f\left(\frac{{\left(x^{2}-m_{j}\right)}^{2}}{\sqrt{x^{2}-{\tilde{x}}^{2}}}\right),\\ f\left(x\right)=\exp\left(x^{2}\right), \end{array}$$

where *y*_*N*_ is the output of the input layer; *j*_*m*_ is the mean of the Gaussian function of the affiliation function layer; *j* is the standard deviation of the Gaussian function of the affiliation function layer; *N* is the output of the neurons of the affiliation function layer.

#### 2.2.3. Third Layer (Rule Layer)

Each neuron in the rule layer is the antecedent part of a fuzzy logic rule, and the neurons in this layer do the product operation on the input signal of that layer. The linear transformation relationship between the input and output of neurons in this layer can be expressed as follows:

(5) $$\begin{array}{c} {\text{net}}^{3}\left(N\right)=\frac{\prod w^{3}x^{3}}{x\left(N\right)},\\ y^{3}\left(N\right)=f\left(\frac{\prod w^{3}x^{3}}{x\left(N\right)}\right)=\exp\left({\text{net}}_{i}^{3}\right),\;i=1,2,3\ldots 9, \end{array}$$

where *y*(*N*) is the output of the affiliation function layer; *w*_*ik*_ is the connection weight value between the affiliation function layer and the rule layer; *y*^3^ is the output of the rule layer.

#### 2.2.4. Fourth Layer (Recursive Wavelet Layer)

**Figure 3:**
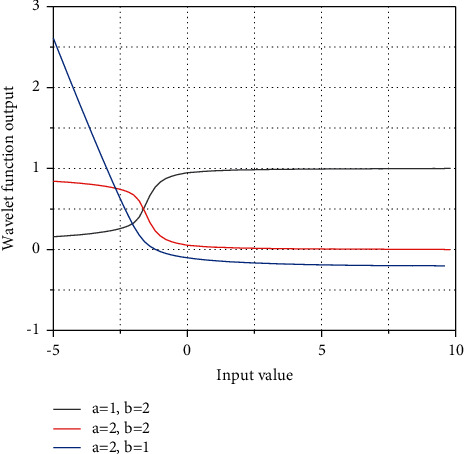
Schematic diagram of the wavelet function.

This layer contains the wavelet function operations, recursive operations, and the posterior part of the fuzzy logic rules. The output of the wavelet function is *k*, denoted as follows:

(6) $$\begin{array}{c} \phi_{ik}=\left(xij\right)=\frac{\left[1-\left({\left(x_{i}-a_{ij}\right)}^{2}/b^{2}\right)\right]}{\sqrt{b_{ik}}}\exp\left(\frac{\left(x_{i}-a_{ij}\right)}{2b_{ij}}\right), \end{array}$$

where *y*_*ik*_ is the *i*-th wavelet function in the *k*-th neuron of this layer; *u*_*i*_ is the output of the *k*-th wavelet function; *w*_*ik*_ is the connection weight of the wavelet function; *a*_*ik*_ and *b*_*ik*_ are the translation and scaling factors of the wavelet function, respectively. From Figure 3, we can see the relationship between different translation and scaling factors on the input and output of the wavelet function.

### 2.3. H-Type Motion Platform to Control Motion Intensity

The physical state is continuously maintained at a low level, and the change in load cannot cause a change in physical state, which may be a sign of overtraining [16]. The long-term accumulation of fatigue leads to the destruction of all the dynamic balance described above, so the stimulation of a short period of load change cannot cause a change in the physical state [17]. Overtraining should also be judged in conjunction with the athlete's mental state and psychological state. In this case, a longer rest period may be required to reestablish the dynamic balance of the physical state in response to the load stimulus.

**Figure 4:**
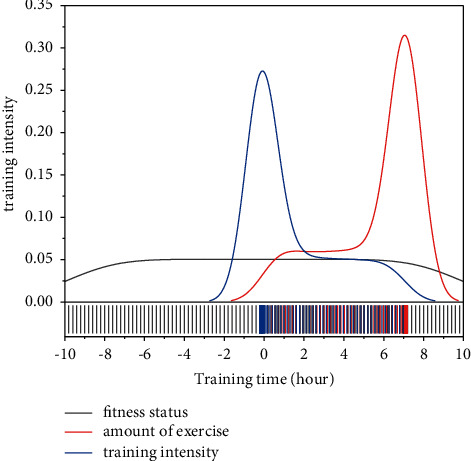
Temporary increase in fitness status due to load reduction after maintaining high levels of load.

The dynamic diagnosis of the athletes' physical state requires continuous testing, which increases the training content of usual training and may interfere with sports training [18]. Therefore, it is significant to study the indicators for rapid testing of physical fitness state and evaluation of load volume on the field [19]. Athletes' responses to training load stimuli have individual characteristics, and a uniform training load may be too high for some athletes and low for others. Therefore, the evaluation of athletes' training load and physical fitness status should be part of daily training, which is convenient for coaches to understand and grasp the changes in athletes' competitive status in time and summarize the effect of each training session and athletes' individual characteristics of load adaptation, as shown in Figure 4. It is possible to predict the output with known inputs or to control the change in the input for a given output.

Even if the evaluation of load and fitness status cannot be normalized and daily, the theory of dynamic diagnosis of fitness status combined with load volume changes has some significance, and its significance lies in the fact that a variety of possibilities should be considered when an athlete's fitness status changes [20]. For example, when an athlete's fitness status decreases, it may be caused by a long-term low level of load stimulation, and the load can be increased, or more likely, the load level exceeds the load that the current functional capacity can bear, and the load should be reduced to recover from fatigue [21]. When the above situation occurs, coaches and athletes will consider a variety of reasons for the change of physical fitness status, combine the change of physical fitness with the change of load, and make a prudent judgment.

## 3. Results and Analysis

### 3.1. Simulation and Results

**Figure 5:**
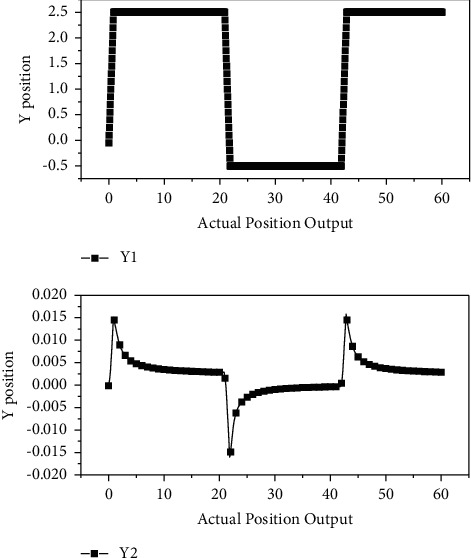
Axis Desired Position vs. Actual Position Output.

The learning rate of the recurrent wavelet fuzzy neural network compensator is set as follows: *ω*_1_ = 0.6, *ω*_2_ = 0.5, *ω*_3_ = 0.4, *ω*_4_ = 0.3, *a* = 0.1, *b* = 1.5, *ω*_5_ = 0.8, *m* = 0.06, *σ* = 1.2.  Figure 5 shows the desired position output curves and actual position output curves of *Y*_1_ and *Y*_2_ axes under the combined action of the global sliding mode controller and the two-axis recursive wavelet fuzzy neural network synchronous compensator, respectively, without load. From the graphs, it can be seen that the actual position output curves of *Y*_1_ and *Y*_2_ axes are basically consistent with the given desired position output curves.

However, all of the above neural networks are feed-forward neural networks, and the input and output of the training samples are only in static mapping because feed-forward neural networks cannot use the information inside the neural network, and the approximation ability of the function is also affected by the training samples. Since most practical applications are dynamic systems, the recursive structure can make use of the internal state of the network to make the information passed this time contains the previous error information, realize the characteristics of dynamic mapping and data storage, and the system can have better dynamic capabilities. Therefore, the recursive wavelet fuzzy neural network compensator designed by combining wavelet fuzzy neural network with recursive structure has better dynamic characteristics in the case of unknown parameters, external interference, and parameter.

**Figure 6:**
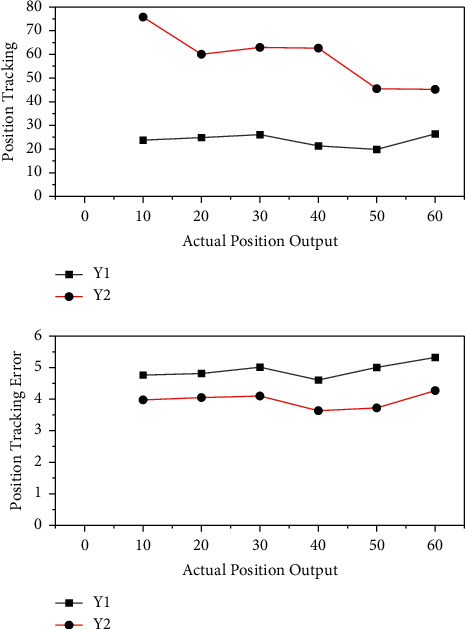
Position tracking error.

The position tracking errors of Y1 and Y2 axis are analyzed by using the global sliding mode control method combined with the recursive wavelet fuzzy neural network synchronization compensator. Based on the results of the above modeling, as shown in Figure 6, it is possible to track the 1.73 changes in the athletes' physical status and the changes in the load and combine the two for dynamic diagnosis. Dynamic diagnosis is not contradictory to the current static diagnosis and can be regarded as an extension of static diagnosis. Static diagnosis can make the horizontal comparison of athletes' physical status, while dynamic diagnosis can make the vertical comparison of athletes' own physical status changes. Dynamic diagnosis combining load and physical status can better judge the load factors of athletes' physical status changes, provide a reference for coaches to scientifically formulate the load amount of training plan, and regulate athletes' status and load.

They attach great importance to the search for indicators established according to the project to establish training characteristics, diagnosis and physical quality combination assessment, physical injury of the athlete, a combination of factors customized training program. In addition, a strict training sequence is needed, for example, premature ageing exercises, other organize core exercises, and then auxiliary exercise strength. For essential strength exercise equipment, the demand of foreign experts has a very high degree of standardization.

The global sliding mode control method combined with the recursive wavelet fuzzy neural network synchronization compensator is used to reduce the dual-axis position synchronization error under the no-load condition. Therefore, using the recursive wavelet fuzzy neural network synchronization compensator designed in this chapter can effectively reduce the synchronization error between the two axes and ensure the tracking performance of the H-type motion platform. At 2 s, the rated load LF = 63 N is applied to the H-type motion stage. The current diagnostic content of the athlete's status mainly includes the diagnosis of sports performance, the diagnosis of competitive ability, and the diagnosis of training load.

**Figure 7:**
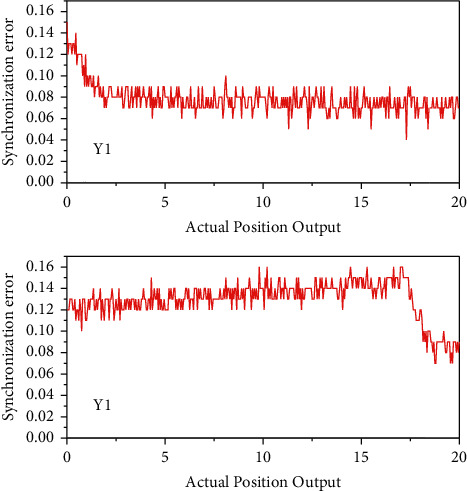
Two-axis position synchronization error.

Under no-load condition, the position tracking errors of Y1 and Y2 axes are analyzed by combining the global sliding mode control method with the recursive wavelet fuzzy neural network synchronization compensator. As can be seen from Figure 7, after adding the rated load, the maximum value of the *Y*_1_-axis position tracking error decreases from 3.59 *μ*m to 1.75 *μ*m compared with the cross-coupled controller, and the maximum value of the regulation process is 2.69 *μ*m; the maximum value of the *Y*_2_-axis position tracking error decreases from 1.65 *μ*m to 2.34 *μ*m compared with the cross-coupled controller, and the maximum value of the regulation process is 2.34 *μ*m. The model refers to the wavelet recurrent fuzzy neural network model, and the variation of the adaptation amount in the model is improved nonlinearly to increase the limit amount of adaptation parameters.

The systems research approach attempts to describe dynamic system processes by reducing them to mathematical models that capture the main influences of the system, abstractly described as a function with a single input and a single output. When modeling the response to exercise training, the organism is considered as a whole system, with the training load as the input to the system and the change in exercise capacity as the output of the system. On the one hand, it is important to define the variable of motor ability based on the results of existing studies, to determine the method of testing, and to collect data according to the definition, and the subject must be required to perform frequent tests throughout the training process and also to simulate the competition environment at full capacity. Acquiring motor ability test data is a big difficulty in the application of the model. Improper testing methods and insufficient data can affect the application of the model. The input to the model is the training load, so the training load must be accurately quantified, and several foreign papers have reviewed methods to quantify the training load.

### 3.2. Real-Time Scheduling of Exercise Intensity

**Figure 8:**
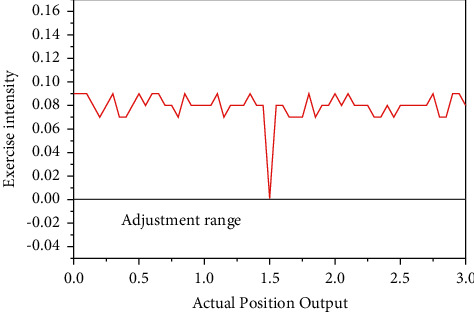
Exercise intensity adjustment range.

Figure 8 shows the two-axis position synchronization error when the global sliding mode control method is combined with the recursive wavelet fuzzy neural network synchronization compensator under the same simulation conditions. As can be seen from the figure, the maximum value of the dual-axis position synchronization error is reduced from 16.78 *μ*m to 1.02 *μ*m compared with the cross-coupled controller, and the maximum magnitude during the regulation process is 15.63 *μ*m. The use of the recursive wavelet fuzzy neural network synchronization compensator designed in this chapter can effectively improve the anti-interference performance of the platform compared with the cross-coupled control compensator between the two axes. The change of athletes' physical state should be seen as a dynamic change process. It is often a review of the athlete at a certain point of time, and the diagnosis of the athlete's physical fitness state should also be a dynamic diagnosis, a continuous observation process, and only continuous observation can reflect the process of the athlete's physical fitness change, and the diagnosis process needs to be combined with the dynamic change of the sport's load because for the athletes with large load training, the change of the load is the main factor affecting the change of the physical fitness state.

Aiming at the tracking problem caused by external disturbances and uncertainties such as nonlinear friction, the single-axis permanent magnet linear synchronous motor is susceptible to the influence of external disturbances and nonlinear friction during the use of the H-type motion stage, the single-axis global sliding mode controller is designed by introducing specific control parameters to weaken the influence of instability in the converging mode phase of the sliding mode control process and effectively suppress the jitter phenomenon of the control system, and then, Liapunov's theorem is used to analyze the convergence and stability of the designed The convergence and stability of the designed global sliding mode control method are then analyzed by using Liapunov's theorem.

**Figure 9:**
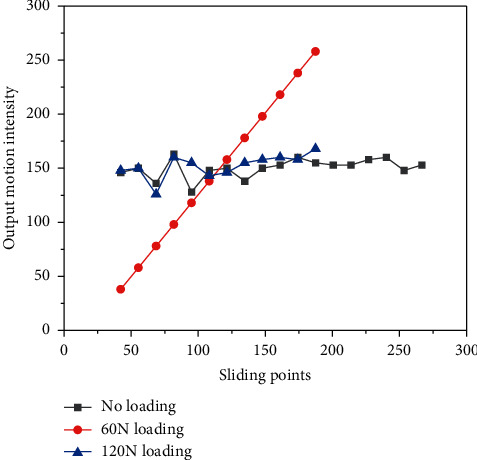
Comparison of controller output motion intensity.

The cross-coupled synchronization controller is designed by combining the single-axis tracking error and the two-axis synchronization error for the two-axis linear motor asynchronous problem.  Figure 9 shows the comparison table of simulation results under the conditions of using different controllers (no loading, 60 N loading, and 120 N loading). According to the control effect, the following conclusions can be drawn: the recursive wavelet fuzzy neural network synchronization compensator designed in this chapter improves the synchronization performance of the dual-axis in *Y* direction compared with the cross-coupling control compensator, and the compensation signal of the dual-axis can be adjusted instantly under the change of the beam load to improve the tracking accuracy of the system single-axis and suppress the effect of the sudden load addition. The system has a better immunity performance. Although the physiological and psychological quality of Chinese basketball players has been developing in recent years, there is still a big gap with Western countries, such as the analysis of current sports problems and possible causes, and in order to improve the competitiveness of professional athletes in promoting physical and mental health, the fund will become the team. Currently, for basketball players, all training is focused on basic physical training and physical training in the training room, while neglecting the importance of postinjury injury prevention training.

The simulation results show that the designed global sliding mode controller can improve the convergence speed of tracking error and ensure the single-axis tracking accuracy of the H-type motion platform compared with the traditional sliding mode controller, and the tracking accuracy and a synchronization accuracy of the system can be further improved after adding the cross-coupled synchronization controller, but the improvement of synchronization control accuracy is not very satisfactory due to the fixed selection of the parameters of the cross-coupled controller. Further improvement is needed.

In order to further improve the synchronization performance of the H-type motion platform, a recursive wavelet fuzzy neural network compensator with online learning capability is designed to replace the cross-coupled synchronization controller between the two axes of the platform, and the parameters in the recursive wavelet fuzzy neural network are adjusted in real time by using the gradient descent method so that the synchronization error of the H-type motion platform can be dynamically compensated according to the change of load. The simulation results show that, compared with the cross-coupled synchronization controller, the recursive wavelet fuzzy neural network compensator can compensate the system input signal in real time, thus effectively reducing the impact of sudden load on the platform single-axis tracking performance and two-axis synchronization error, and reducing the impact of uncertain disturbance terms on the system control process, and improving the synchronization performance and robustness of the direct-drive H-type motion platform.

## 4. Conclusion

In this paper, the model refers to the wavelet recurrent fuzzy neural network model, and the variation of the adaptation quantity in the model is improved nonlinearly by increasing the limit quantity parameter of adaptation. The data required for parameter estimation were the actual training data of six outstanding basketball athletes (average age 18.2 ± 0.75, average training years 4.6 ± 0.49), and the coaches' training program was not intervened during the test. In order to further reduce the biaxial synchronization error of the sports platform and improve the stability of the system, the wavelet transformation capable of time-varying signal analysis and the recursive structure with dynamic capability were combined with the fuzzy neural network, and the learning ability of the neural network was used to learn and adjust the scaling and translation factors in the wavelet function, the mean and standard deviation in the fuzzy structure, and the connection weights between the layers, according to the biaxial synchronization. The simulation results show that the designed global sliding mode controller can improve the convergence speed of tracking error and ensure the single-axis tracking accuracy of the H-type motion platform compared with the traditional sliding mode controller, and the tracking accuracy and a synchronization accuracy of the system can be further improved after adding the cross-coupled synchronization controller, but the improvement of synchronization control accuracy is not very satisfactory due to the fixed selection of the parameters of the cross-coupled controller. Further improvement is needed.

## Figures and Tables

**Table 1:** Model parameters.

Individual athletes	Credibility	Distinguishability	Number of cases
1	0.23	0.14	2
2	0.64	0.15	4
3	0.96	0.16	2
4	0.33	0.04	4
5	0.69	0.12	5
6	0.21	0.06	7
7	0.62	0.08	6

**Table 2:** Factors affecting exercise capacity.

Influencing factors	Number of cases	Impact cases	Impact score
Physical fitness	5	20.11	15.32
Weather	5	19.48	15.24
Opponent	5	20.16	14.33
Age	3	13.95	10.65
Diet	4	18.73	14.26
Coaches	59	61.88	60.36
Teammates	6	19.78	16.09
Training	15	27.22	22.25

## Data Availability

The data used to support the findings of this study are available from the corresponding author upon request.
